# An architecturally constrained model of random number generation and its application to modeling the effect of generation rate

**DOI:** 10.3389/fpsyg.2014.00670

**Published:** 2014-07-01

**Authors:** Nicholas J. Sexton, Richard P. Cooper

**Affiliations:** Department of Psychological Sciences, Centre for Cognition, Computation and Modelling, Birkbeck, University of LondonLondon, UK

**Keywords:** random number generation, executive function, cognitive control, cognitive architecture, computational model, supervisory system

## Abstract

Random number generation (RNG) is a complex cognitive task for human subjects, requiring deliberative control to avoid production of habitual, stereotyped sequences. Under various manipulations (e.g., speeded responding, transcranial magnetic stimulation, or neurological damage) the performance of human subjects deteriorates, as reflected in a number of qualitatively distinct, dissociable biases. For example, the intrusion of stereotyped behavior (e.g., counting) increases at faster rates of generation. Theoretical accounts of the task postulate that it requires the integrated operation of multiple, computationally heterogeneous cognitive control (“executive”) processes. We present a computational model of RNG, within the framework of a novel, neuropsychologically-inspired cognitive architecture, ESPro. Manipulating the rate of sequence generation in the model reproduced a number of key effects observed in empirical studies, including increasing sequence stereotypy at faster rates. Within the model, this was due to time limitations on the interaction of supervisory control processes, namely, task setting, proposal of responses, monitoring, and response inhibition. The model thus supports the fractionation of executive function into multiple, computationally heterogeneous processes.

## Introduction

In a random number generation (RNG) task, subjects are instructed to randomly produce items from a given response set (e.g., single-digits 0–9), as if selecting and replacing items from a hat. The task typically requires subjects to produce a sequence of arbitrary length (e.g., 100 digits). Despite its apparent simplicity, the task is surprisingly complex, and appears to require the co-ordination of a diverse set of cognitive processes in order to generate an apparently random sequence.

When human-generated sequences are analyzed according to information-theoretic criteria for randomness, performance is typically poor, with such sequences exhibiting a number of substantial biases. Perhaps surprisingly, however, such biases show considerable regularity and robustness across subjects and modalities. For example, subjects strongly avoid consecutive repetitions (e.g., Baddeley et al., 1998; Towse, 1998), even though in ideally random sequences of sufficient length, such repetitions occur with a non-negligible expected frequency—for a response set of 10 items, approximately one-tenth of responses should be consecutive repeats.

One possibility is that participants are poor at generating random sequences because they have an erroneous concept of randomness^1^, an inability to perceive whether one's own sequence is sufficiently random, or both (see Bar-Hillel and Wagenaar, 1991, for a review, but see Nickerson, 2002 for a critical treatment). This may well contribute to a normative, base-level bias, but the biases shown by participants are reliably modulated by experimental manipulations (e.g., concerning the rate of production, the concurrent performance of different secondary tasks, or the response modality). Additional factors therefore appear to underlie the biases shown across experimental conditions. In fact, an analysis of the production mechanisms involved in RNG suggests the involvement of multiple, functionally separable but interdependent processes. For example, Baddeley et al. (1998) propose that RNG involves, minimally: maintaining and updating a representation of previous responses; the selection of responses from an internal representation of the response set; and the suppression of habitual sequences such as counting. The involvement of multiple heterogeneous processes is supported by evidence from behavioral experiments (e.g., Baddeley et al., 1998; Towse, 1998; Cooper et al., 2012), TMS (e.g., Jahanshahi et al., 1998), neuroimaging (Jahanshahi et al., 2000), and individual differences studies (e.g., Miyake et al., 2000) and there is an apparent consensus that these processes correspond with putative executive functions, including working memory monitoring/updating, task setting, and response inhibition.

While such executive functions have been extensively studied in numerous experimental paradigms, and specified in computational terms (e.g., Gilbert and Shallice, 2002; O'Reilly and Frank, 2006; Boucher et al., 2007; Brown et al., 2007), modeling has tended to focus on simple experimental tasks intended to isolate or decompose single executive functions. Computational accounts of more complex tasks which seek to show how more complex behavior is regulated as a synthesis of a number of such executive functions operating in concert have been infrequent (although, see Sood and Cooper, 2013).

The present article presents a model of the RNG task within a theoretically motivated executive function architecture, and evaluates the model against existing empirical data sets. Of the remainder of this paper, the Measures of Randomness and Behavioral Effects section reviews how biases in random sequences have been assessed and how they have variously been manipulated in behavioral experiments. The Existing Models of RNG and their Limitations section discusses cognitive theories of RNG. In An Architecturally Grounded Model of RNG, we present a computational model of the task, grounding the model within the specification of a complete, task-general executive architecture. The Simulations section presents four simulations in which the model performs RNG at various rates. Together, the simulations demonstrate how some of the characteristic effects of modulating generation rate in human subjects might be accounted for in terms of an automatic contention-scheduling system which produces habitual behavior, regulated by rate-limited supervisory processes. The Results section presents simulation results. The Discussion section presents a general theoretical discussion of the simulations and the presented architecture. The Supplementary Material contains implementational details and parameters for the model^2^.

## Measures of randomness and behavioral effects

The randomness of a sequence may be assessed in a number of ways. In an ideally random sequence of infinite length, the overall frequency of responses should be equal. Biases away from such equality are commonly assessed using R, an information theoretic index of redundancy which is derived from the number of occurrences of each response (Baddeley et al., 1998). However, a sequence that simply cycles repetitively through the response set would achieve minimal redundancy whilst being entirely predictable, suggesting that additional criteria are required. One alternative is the RNG score (Evans, 1978), which measures the relative usage of all consecutive two-response combinations (digrams), which should also be equal in an infinitely long, ideally random sequence. Hence, a higher RNG score can be thought of as reflecting overall overuse of certain digrams. While the RNG score gives an overall assessment of sequential bias, other indices are sensitive to specific stereotyped sequences. For example, when using a familiar response domain such as letters (e.g., Baddeley, 1966) or digits (e.g., Jahanshahi et al., 1998) it appears that subjects must inhibit overlearned continuations, such as cycling responses in alphabetical order, or counting, and a number of metrics have been used to specifically assess such habitual sequence biases. Adjacency (A), is simply the proportion of responses that reflect adding or subtracting one to the previous response (±1 associates), while count scores (CS1, CS2) are weighted to the length of a subsequence, and defined respectively as the summed square of the length of each sequence that consists of ±1 or ±2 associates (Spatt and Goldenberg, 1993). Such measures do not assess the regularity with which responses are re-used. The distribution of the gaps between successive instances of the same response may be calculated, and the average (usually, the median) repetition gap (RG) assesses the tendency of subjects to cycle through the response set with a greater predictability than ideally random sequences (Towse and Neil, 1998). Thus, no single metric provides a complete assessment of randomness.

A large number of indices may be calculated from an arbitrary sequence, and compared with the expected values associated with true randomness (generally, calculated empirically through repeated computer generation of pseudorandom sequences or random number tables) for an assessment of bias. A crucial question is the extent to which indices of randomness dissociate. When such varied metrics are obtained from written sequences generated by human subjects, a factor structure is found that shows individual differences in human biases different to that from ideally random sequences of the same length. Ginsburg and Karpiuk (1994) suggested a three-factor model of subject biases, including factors they named Cycling (loading primarily on median RG), Seriation (with strong factor loadings on indices of adjacent responses, similar to CS1 and A, and unequal digram usage, similar to RNG), and Repetition (with strongest factor loading on the number of consecutive repetitions). This suggested that underlying the biases evident in randomness indices is a consistent structure, relating to the operation of various underlying cognitive processes, the efficacy of which differs across individuals.

Subsequent factor analyses of verbal responses to an RNG task (e.g., Towse and Neil, 1998; Miyake et al., 2000) confirmed these findings, although with an additional factor, Equality of Response Usage, which loaded primarily on redundancy (R). Miyake et al. hypothesized that the suppression of prepotent responses (identified as Seriation by Ginsburg and Karpiuk, 1994) requires response inhibition, while equality of response usage requires the updating of memory representations of previous responses. Indeed, relating variation on the Equality of Response Usage factor to differences in working memory updating is consistent with the absence of such a factor in the analysis of Ginsburg and Karpiuk (1994) which, being based on a written version of the task, obviated the need for subjects to use memory to regulate response equality. Miyake and colleagues found the best fitting structural equation model indeed linked the two factors to latent variables which putatively indexed the respective executive functions, as derived from a selection of simple executive tasks.

Miyake et al.'s study provides additional support for the hypothesis that different indices of randomness do indeed index different control functions (rather than, for example, different conceptions of randomness). It is difficult to see why individual differences in biases underlying random generation should correlate with latent factors identified with cognitive control functions, if biases in random generation are merely due to limitations in the ability to perceive randomness. Additionally, the study suggests plausible candidate functions underlying two of the main dimensions of bias in random generation performance. However, it has a number of limitations: the data are purely correlational; the fit of the authors' favored model (with single path from each latent variable to randomness factors) is only marginally better than the fit of several alternative models; and the model does not provide a mechanistic account of task performance in terms of cognitive processes (see Cooper et al., 2012, for a more detailed discussion).

Consistent with these findings from individual differences methodologies, experimental studies have found that various indices of randomness are differentially sensitive to behavioral manipulations. One commonly reported finding is the effect of varying the rate of response generation, with an increase in generation rate producing an increase in the tendency to use counting as a stereotyped strategy (indexed by CS1 or A scores). It appears that at faster rates, subjects have difficulty suppressing overlearned, habitual sequences (Towse, 1998; Jahanshahi et al., 2000). In a PET study, the left dorso-lateral prefrontal cortex (LDLPFC) appeared to be critical for such suppression of habitual sequences, with the region more active when subjects performed RNG compared to counting (Jahanshahi et al., 2000). Furthermore, activation in the region appeared to be related to a behavioral measure of stereotypy, with the faster rates of generation producing an increase in CS1 scores and a decrease in LDLPFC activation. Jahanshahi and colleagues interpreted this as reflecting the breakdown of this region's ability to suppress habitual responses at fast rates of generation. This conclusion was supported by findings from a second study using Transcranial Magnetic Stimulation (TMS) over the region, which produced a similar increase in CS1 without having a significant effect on other randomness indices for equality of response usage (*R*) or cycle length (*RG*) (Jahanshahi et al., 1998). These results are paralleled by neuroimaging findings from another generation task—verbal fluency—in which the same region was active for word production, but not word comprehension (Frith et al., 1991). Taken together, these findings suggest that the role of the LDLPFC in generation tasks is not specific to random sequences, but instead related more generally to response selection and the suppression of bias. This evidence appears incompatible with an explanation of random generation bias purely in terms of an erroneous conception of randomness, and again points to a control-process account of random generation.

Varying other task parameters has tended to modulate alternative indices of randomness. Varying the size of the response set, for example, appears to affect the subject's ability to achieve equal use of each element of the response set, although this effect disappears in modalities where the responses are represented visually, or when a visible representation of the response set is provided (Towse, 1998). From this evidence, it appears as though equal use of the response set is dependent on the subject representing the response set, either externally, or internally, where the ability to keep track of previous responses appears limited by memory constraints.

An alternative approach to the investigation of the cognitive processes underlying random generation has been to use a dual-task methodology, based on the assumption that the performance of a simple task should lead to performance impairments on a concurrent complex task through a reduction in the capacity of cognitive processes which are shared between the tasks. Specifically, the findings of Miyake et al. (2000) suggest that concurrent performance of a simple response inhibition task should increase prepotent associates bias, while a concurrent memory monitoring/updating task would increase equality of response usage bias. Inconsistent with this prediction, however, in a visual/spatial version of the task, Cooper et al. (2012) found prepotent associates indices (including RNG and A) were most biased when RNG was paired with a 2-back task (held to primarily involve working memory monitoring/updating), while equality of response usage (R) was affected both by concurrent performance of a 2-back task and a digit-switching task (held to require task switching).

In summary, while an erroneous conception of randomness might explain the existence of normative bias, such limitations are inadequate to explain findings from three main strands of evidence. Firstly, individual differences studies suggest that biases are regular across subjects, and appear to correlate with putative executive functions. Secondly, neuroimaging and TMS data suggests that the degree of bias in a sequence is modulated by activity in a specific region of prefrontal cortex, which is also linked to response production, but not perception, in a generation task which does not involve randomness. Thus, the control processes involved in controlling bias appear more general than would be suggested by specific limitations in a concept of randomness. Thirdly, a limited concept of randomness cannot explain why biases are modulated by behavioral manipulations (e.g., generation rate, concurrent performance of a secondary tasks) or procedural variations (e.g., response modality, response set size), in such a way as to produce qualitatively different patterns of behavior^3^. While the evidence reviewed thus far is consistent with the hypothesis that random generation performance requires multiple co-ordinated executive functions, it does do not yet present a coherent picture of how the cognitive processes interact during task performance, or which biases reflect which executive functions. One aim of the present work is to present such a theoretically-motivated account.

## Existing models of RNG and their limitations

Baddeley et al. (1998) suggested that the process of random generation can be understood by analogy with other generation tasks, such as category generation. In such a task, subjects typically make use of strategies or schemas to generate a cluster of related items (e.g., for the category “animals,” typical schemas might include “household pets” or “farmyard animals”), switching schemas when they cease to produce novel responses. Random generation is held to involve similar use of retrieval schemas. In the case of digit generation, schemas might include habitual schemas such as counting or reciting telephone numbers; or deliberate strategies such as iteratively subtracting two. However, in contrast to category generation, to comply with the requirement that the sequence appear random, subjects are required to frequently switch between different generation schemas. According to this model, distinct cognitive processes are responsible for monitoring responses before they are generated, inhibiting conspicuously non-random responses, and switching between alternative generation schemas. The schema switching process must be capacity limited, or ideal random generation could be achieved by deliberate switching of schemas on every response. Impairment in performance could result from three possible sources: the availability and capacity to switch between retrieval schemas, the capacity to monitor and inhibit prior plans or responses if they would produce an insufficiently random output, and the capacity to maintain a mental representation of previously used schemas. The processes of monitoring, inhibition, and schema switching were ascribed to the central executive of the multicomponent working memory model (Baddeley, 1986, 2000).

Jahanshahi et al. (2000) proposed a similar model, also based on extrapolating the processes thought to be involved in category fluency. In this model, an associative network represents response alternatives as a series of nodes, with node activation spreading through mutual, variably weighted excitatory connections. The strongest weights are between neighbor pairs (representing ±1 associates) with the next strongest between alternate pairs, such that the unrestrained output of the network resembles counting. An active controller comprises multiple control processes, including inhibitory modulation of response alternatives, monitoring output to detect violations of task rules, and access to information in long term memory (e.g., task rules, a subjective conception of randomness, etc.). The authors propose that the mechanism for the control of responses is the active inhibition of a prior response's neighbor nodes (or strongest connections), to suppress inappropriate, habitual responses. Inability to produce a random sequence is, as in Baddeley et al. (1998), due to capacity limitations of the active controller, in particular at the fastest rates of generation.

One limitation of the models of Baddeley et al. and Jahanshahi et al. is that neither is fully specified in computational terms. The main aim of the present article is therefore to present a computationally explicit account of the contribution of specific control functions to the generation of random numbers by human subjects. This approach offers advantages beyond the above verbal models. It makes explicit how varied randomness indices are dependent on the interaction of specific control processes; and it shows how the interaction of such control processes might be affected by task factors such as generation rate.

## An architecturally grounded model of RNG

While it would be possible to develop a computationally complete model of RNG based on the above verbal description, there is much to be gained by developing the model within the more constrained theoretical framework of a cognitive architecture. A cognitive architecture specifies at a gross level a theory of the organization of the processing subcomponents that make up a cognitive system (in this case, the human cognitive system). By developing a model within an architecture, the model inherits constraints from the architecture (Newell, 1990) and when models of a range of tasks are developed within a single architecture, constraints from the full range of tasks can help to further specify elements of the architecture, thereby effectively resulting in additional constraints on each specific model (but see Cooper and Shallice, 1995).

At the time of writing, the most well-developed cognitive architecture is the ACT-R (Adaptive Control of Thought—Rational) architecture of Anderson and colleagues (e.g., Anderson, 2007), though there are a range of proposals in the literature (including most notably Soar, e.g., Newell, 1990; Laird, 2012; and EPIC, Meyer and Kieras, 1997). Here we present a novel architecture—the Executive Subprocess (ESPro) architecture—which is based on theories of the organization of the “central executive” (Baddeley, 1996) and the break-down of executive functioning following neurological damage (e.g., Norman and Shallice, 1986; Shallice et al., 2008). We return to the relation between the ESPro architecture and other extant cognitive architectures in the general discussion.

### The executive subprocess (ESPro) architecture (version 1.0)

**Figure 1:**
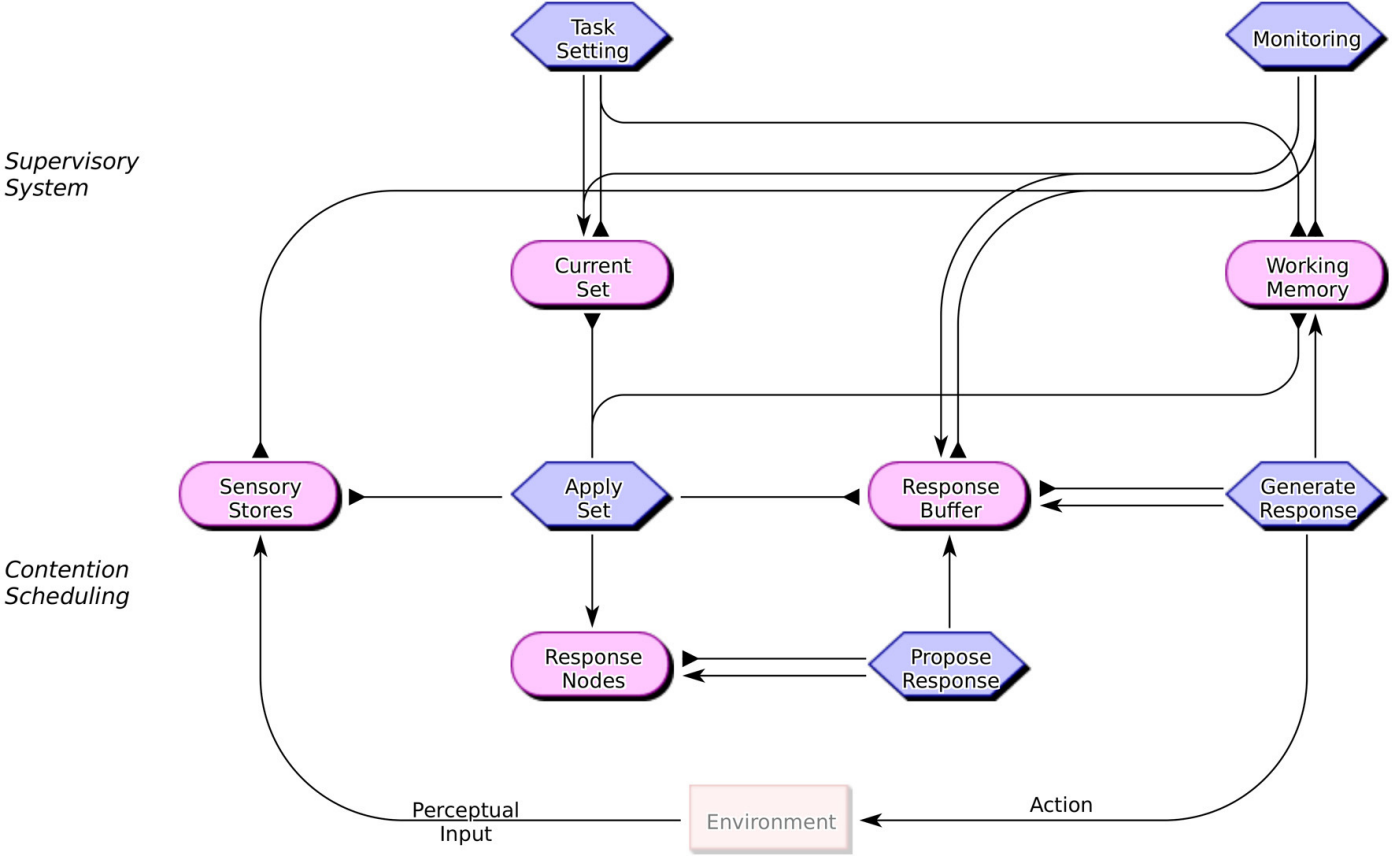
**Components of the Executive Subprocess (ESPro) architecture**. Hexagonal boxes represent processes that generate output while rounded rectangles represent buffers that store representations (possibly with associated activations). Pointed arrows between boxes show message passing. Arrows with flat heads represent the reading of information from buffers by processes.

Figure 1 shows the components of the proposed ESPro architecture (version 1.0) and their potential interactions. The figure adopts the conventions of the COGENT modeling environment (Cooper and Fox, 1998; Cooper, 2002), whereby a cognitive system is decomposed into interacting components (generally buffers, shown as rounded rectangles, and processes, shown as hexagonal boxes), with interactions shown by different types of arrow (for sending messages to processes or querying the content of buffers). In order to develop a fully operational computational model from a COGENT diagram it is necessary to specify the properties of the component buffers (e.g., whether and how capacity limitations and decay might operate) and the rules of operation of the component processes.

ESPro assumes a basic distinction between routine or over-learned behavior and non-routine behavior (see Norman and Shallice, 1986). The former is assumed to be the product of a schema-based system (referred to as *contention scheduling* and shown in the lower portion of Figure 1) while the latter arises from the modulation of that schema-based system by higher cognitive processes (referred to collectively as the *supervisory system* and shown in the upper portion of Figure 1). Many of the components of the ESPro architecture have a generic, task independent, operation. Consider first the contention scheduling components. Current Set is a buffer that contains a representation of the task set or schema that, at a given point in time, controls behavior. (In the case of a random generation task this might be a counting schema.) Apply Set operates by applying the schema contained within Current Set to the current stimulus (which is assumed to be contained in the Sensory Stores), potentially drawing upon information stored in Working Memory and Response Buffer. The effect of this is to activate a representation of a potential response within Response Nodes. Propose Response monitors the activation of elements in Response Nodes, and when one exceeds a selection threshold the corresponding item is copied into Response Buffer. Generate Response monitors Response Buffer. When it detects a putative response it generates it (e.g., articulating the word or pressing the corresponding response key) and stores the response thus generated in Working Memory. These components thus implement a system for the generation of habitual or schema-driven behavior.

The operation of Apply Set is based on the contention scheduling system described by Norman and Shallice (1986), see also Cooper and Shallice (2000). It assumes that routine behavior involves application of behavioral routines (schemas) to perceptual input (i.e., the contents of Sensory Stores). Responses generated in this way must be stored in Working Memory (or some short-term buffer) as many routine sequential tasks consist of actions that are dependent on previous actions, even when those previous actions have no persistent sensory consequences. For example, when adding sugar to a hot beverage it is necessary to record in some way when sugar has been added as once the sugar has dissolved it cannot serve as a perceptual cue. Similarly in the case of RNG it is necessary to record, at least temporarily, each response, as schemas such as counting need to know the previous response in order to generate the next putative response.

Empirical and neuroimaging studies involving tasks such as the stop signal and Go/NoGo tasks (e.g., Verbruggen et al., 2006, 2008; Swick et al., 2011) and the task switching paradigm (e.g., Philipp et al., 2007) imply that response selection and response generation are separate processes. This is reflected within the ESPro architecture by the distinction between the Propose Response and the Generate Response processes. Once a response is proposed, it is temporarily stored in the Response Buffer prior to being generated. A putative response can then be withdrawn or withheld prior to generation if supervisory processes (specifically Monitoring) determine that the response is inappropriate in the current context.

The remaining components of the architecture allow for the modulation of routine behavior in non-routine situations. Monitoring detects when a proposed behavior fails to meet task requirements^4^. In the case of random generation this process must detect when a putative response is “insufficiently random,” as described below. When Monitoring detects such a failure, it inhibits production of the proposed response and triggers generation of a more appropriate response. It does the latter by inhibiting the current schema (i.e., the contents of Current Set). This in turn triggers Task Setting to generate and install an alternative schema in Current Set. Routine operation (via contention scheduling) may then resume, with the alternative schema (hopefully) leading to the proposal of a more appropriate or acceptable response.

### A model of RNG within the ESPro architecture

Applying the ESPro architecture to a specific task such as random generation requires specifying the operation of the two key task-specific components of the architecture: Task Setting and Monitoring. The role of Task Setting is to propose and instate a new schema or task set whenever the process detects that there is no schema currently controlling behavior (i.e., Current Set is empty). The operation of the process depends on the available schemas (which is task specific), and in highly complex tasks the process may involve problem solving and planning. This level of complexity is not required, however, for the random generation task, and in the current model we explore the assumptions that (a) the available schemas may be effectively approximated by a set of schemas that can transform (by addition or subtraction of a fixed quantity) any response into any other response, and (b) Task Setting selects from these schemas stochastically, with “repeat response” having a much smaller probability of selection (e.g., of 0.01, assuming a response set size of 10) than all other schemas, which have equal probability of selection (e.g., of 0.11, given the response set size of 10). Thus, Task Setting implements a bias away from selection of the schema supporting repeat responses, but beyond this it is not biased in its schema selection.

Within the general architecture, the Monitoring process is required to detect when a planned response (i.e., a putative response that has been entered into Response Buffer) is inappropriate, and hence should be inhibited in favor of a more considered response. In the context of the random generation task, Monitoring must determine when a putative response is in some sense insufficiently random. On detection of such a putative response it should be removed from the Response Buffer and the schema that led to its generation should be deselected (i.e., removed from Current Set). Presumably there are individual differences in what one considers “insufficiently random.” For the purposes of the current simulation we assume that criteria for rejection of a response can be abstractly represented with a simple set of if/then rules. Specifically, the model treats a putative response as insufficiently random in the event of any of three conditions: (a) the model can recall having recently produced the response (i.e., there is a record of it in Working Memory); (b) the response is one more or one less than the previous response; or (c) the response together with the previous two responses forms a sequence with equal intervals between each (e.g., 2, 5, 8 or 9, 5, 1). These three rules can be seen as implementing an avoidance of repeat responses, counting, or repeated use of a single schema, respectively, and are thus consistent with the verbally specified models of Baddeley et al. (1998) and Jahanshahi et al. (2000).

The only further details required to specify the model of random generation concern the behavior of the Working Memory and Response Node buffers^5^. Working Memory is a short-term store that must be capable of holding an ordered set of recently generated responses. The information in Working Memory is used by Apply Set to generate a putative response by applying the current schema (stored in Current Set) to the recollection of the previous response (as recovered from Working Memory), and by Monitoring to check whether a putative response, once generated, is sufficiently random (as above). The model is agnostic about how the short-term storage aspects of Working Memory are achieved. Thus, the buffer might have limited capacity (such that when capacity is exceeded older elements are over-written) or decay might operate on its contents (with the likelihood of an element becoming inaccessible increasing with time or with the number of elements stored in the buffer). In the simulations reported below we consider two distinct implementations of Working Memory.

The Response Node buffer is an interactive activation network with one node for each possible response. Nodes compete for activation according to standard interactive activation principles with lateral inhibition between nodes ensuring that no more than one node is highly active at any time. Apply Set operates by exciting the node corresponding to the favored response, but this is modulated by associative excitation within the network (so if the node for “3” is active it will excite the nodes for its closest associates, “2” and “4”). Propose Response detects when a node's activity exceeds a threshold. A representation of that response is then placed into Response Buffer and the corresponding node in Response Nodes is inhibited. Activation dynamics is governed by the interactive activation equations of Cooper and Shallice (2000) with a threshold of 0.80.

### A worked example

An example of processing in the model is as follows. Let us assume that Working Memory contains three previous responses, 0, 2, and 5, while Current Set contains the schema +3. At this stage, the most active response node is 6, due to the spreading of activation from the previously active node, 5, and the activation of this node laterally inhibits all other response nodes. However, within the contention scheduling system, Apply Set excites the 8 node on each cycle, as a result of applying the +3 schema to the previous response in working memory (5). Activation of the 6 and 8 nodes is competitive, and in this example, the 8 node “wins,” crossing an activation threshold first. Propose Response then creates an instance of the “8” response in the Response Buffer and correspondingly inhibits the “8” Response Node.

Once a response has been added to the Response Buffer, the model is ready to produce it at any appropriate time. In the model, this time is given by the number of cycles specified by the Generation Rate parameter. In the interim, however, the contents of the Response Buffer are open to inspection by Monitoring. To continue our example, Monitoring matches the “8” response against the contents of working memory, according to one of its rules specifying an insufficiently random response (in this instance, reflecting repeated usage of the +3 schema) and performs two actions: firstly, inhibiting the activation of the “8” response within the Response Buffer^6^, secondly, clearing the schema from *Current Set*. Next, Task Setting sets a new schema (−2) which Apply Set uses to excite the “3” response node. Meanwhile, the “6” response has remained relatively active from the earlier stages of processing, and crosses the activation threshold first, and is transferred to the Response Buffer.

This cycle of proposing responses, monitoring, task-setting, and proposing new responses continues until a either a response is found which does not trigger any Monitoring rules, or the model runs out of time and is forced to generate whatever response is in the Response Buffer (i.e., in our example, if the generation period expired here, the next response to be generated would be “6”). Thus, at longer rates of generation, the model may proceed through more iterations, and is more likely to generate a response fully consistent with its concept of randomness.

## Simulations

### Model implementation

A consistent finding of empirical studies of RNG is that varying the rate of generation leads to a substantial decrease in randomness at the fastest rates of generation, characterized by an increased tendency to employ the simplistic, overlearned strategy of counting. This effect is held to be the result of a breakdown in control processes underlying random generation (Baddeley, 1996; Towse, 1998). Why might this occur at faster rates of generation? We note two distinct possibilities. Firstly, a breakdown may be directly related to rate of generation; for example, if the elimination of stereotyped responses depends on temporally extended supervisory processes, increased bias may be a direct result of limited processing time. Alternatively, the breakdown of control processes may be indirectly related to generation rate via an increase in generic cognitive load, or a reduction in the availability of specific cognitive resources, as a result of the increasingly demanding nature of the task at faster rates of generation. Although the present model architecture could be adapted to represent either proposal, the following simulations explore the behavioral implications of the first, more parsimonious, hypothesis: that decreased randomness and increased sequence stereotypy is a direct result of time limitations on supervisory processes.

While certain aspects of the model, such as the architecture of the supervisory system, represent core theoretical claims; other components are included only to provide a complete, executable, computational model. We argue that the precise instantiation of working memory is such an implementational detail, and that a range of implementations of working memory would produce a broadly similar qualitative pattern of behavior. To substantiate this argument, two alternative implementations of working memory are presented (simulations 1A, 2A vs. 1B, 2B). Additionally, two alternative implementations explore how the effect of generation period on one specific dependent variable, RNG, may arise from individual differences in schema selection biases (simulations 1A, 1B vs. 2A, 2B). Table 1 shows the four sets of simulations.

**Table 1:** **Overview of simulations**.

		**Working memory implementation**	
		**Functionally abstract**	**ACT-R—inspired**
**Schema selection**	Equiprobable (identical subjects)	Simulation 1A	Simulation 1B
	Idiosyncratic (variation between subjects)	Simulation 2A	Simulation 2B

#### Working memory implementation

While a wide range of theoretically-motivated models of working memory have been proposed (for a review, see Miyake and Shah, 1999) the present implementation of the RNG model aims to avoid commitment to any specific implementation. Thus, two alternative implementations of the model are presented, offering a functionally abstract working memory system (Simulations 1A and 2A) and a theoretically-driven implementation adopting a number of the assumptions and features of the ACT-R architecture (Simulations 1B and 2B).

***A functionally abstract implementation of working memory***. For the purposes of simulations 1A and 2A, working memory is implemented as a buffer which contains a temporally-ordered list of representations of previous responses. This implementation differs from the standard buffers available within the COGENT framework^7^, in that it is assumed that the likelihood of a representation decaying is a function of its position in the buffer, rather than the elapsed time since it was added.

This assumption is based on empirical findings that repetition suppression appeared to occur as a function of the number of intervening items, orthogonal to generation rate, and not directly related to the amount of time that had elapsed since its production (Towse, 1998), in contrast to findings on the recency effect in typical working memory paradigms (e.g., Page and Henson, 2001). Indeed, the assumption that the decay of memory for previous responses is orthogonal to generation rate is critical to model performance. Possible mechanisms for this phenomenon are briefly discussed in the following section.

In implementational terms, working memory implements the assumption that a normalized activation level is distributed across the contents of working memory, with new items in working memory “stealing” activation from existing items such that an item's activation is approximately inversely proportional to its position in the buffer. This intuitive description is formalized in Supplementary Material (Working memory activation equations).

***An implementation of working memory inspired by ACT-R***. While the previous implementation aims to abstract away from the algorithmic detail of working memory as a modeling convenience, it may be argued that its implementation is overly simplified and lacks theoretical motivation. It implements memory representations as all-or-none—if they are present in the buffer, their instantaneous retrieval is guaranteed; however on decaying they are irretrievably lost. It might be argued that it is implausible that a monitoring process accesses the most recent and most distant items in working memory equally easily; in demanding working memory tasks requiring continual updating such as the N-back task, for example, cognitive load increases as more items are required to be maintained in working memory (Fletcher and Henson, 2001). An alternative implementation of working memory aims to demonstrate that the behavior of the model is not dependent on the simplified working memory assumptions made in simulations 1A and 2A. Here, we assume that retrieval of older items in the buffer should take more time. Hence at faster rates, the monitoring system will be restricted to checking responses against fewer previous responses in working memory.

One available implementation of this idea is that used by ACT-R to model retrieval from declarative memory (e.g., Anderson, 2007). Within the ACT-R architecture, memory representations are activated portions of long-term memory, with the activation value decaying over time. Thus, in contrast to the previous implementation, items do not decay from working memory—each possible response is represented once. The probability of successfully retrieving memories, and the time required for retrieval, are functions of an item's activation. Adopting these assumptions offers some independent, theoretically validated, constraints for working memory, while testing the claim that the specific implementation of working memory is incidental to its qualitative behavior.

Our implementation of activation in working memory departs from the ACT-R algorithms in one important respect. We assume, for the purposes of retrieval latency and probability equations, that activations of working memory items are relative to mean activation in the buffer, rather than absolute values, in order to ensure working memory decay is independent of generation rate, and so as to stabilize the capacity of working memory between the start and end of the task. This additional assumption is similar to that adopted in the functionally abstract implementation of working memory above. However, in the canonical implementation of base-level learning in ACT-R, successful retrieval of chunks in memory increases their level of activation, a mechanism not implemented in the present model. If slower rates of generation permit more memory retrievals as a by-product of more thorough monitoring, it seems reasonable that this mechanism might counterbalance decaying activation to produce WM decay that is orthogonal to generation rate, as has been assumed here. Future experimental and computational work might fruitfully explore whether working memory decay is indeed orthogonal to generation rate in the RNG paradigm, and whether this can be accounted for by models which propose active maintenance as a by-product of monitoring, or a dedicated process (e.g., verbal rehearsal), although such work is outside the scope of the present study and these points will not be discussed further.

#### Schema selection

In the model, schemas represent a probability distribution of all potential responses, given a previous response. For simplicity, however, simulations 1A and 1B essentially model the performance of a single subject, where the selection of all non-repeat schemas by Task Setting is equi-probable. In human subjects, however, it is plausible that idiosyncratic schema biases are superimposed over shared schemas such as counting, or counting in twos. This creates issues for empirical assessment of bias. On one hand, certain indices of randomness, such as associates scores, reflect population averages (rather than biases evident in any single individual) and thus may not be sensitive to such between-subject variability. On the other hand, measures of bias which are calculated individually for each subject, such as the RNG score, may reflect idiosyncratic bias that is not evident in associates scores.

Simulations 2A and 2B therefore model a varied population of subjects. A unique set of biases is created for each modeled “subject” by scaling each element of the original schema selection weights matrix with a noise value obtained by sampling from a Gaussian distribution. We predicted that this will increase individual-specific biases, indexed by RNG scores, without substantial changing mean associate scores (but producing an increase in their variances).

### General simulation methods

#### Simulation procedure

For each of the four simulation studies, 36 virtual subjects produced a sequence of 100 responses from the set 0 to 9 (inclusive) for each of six experimental conditions. All sequences were scored on the standard dependent measures described above, namely CS1, CS2, R, RNG, and RG. The model includes several parameters that are essential to its correct operation (e.g., spreading activation in the response nodes) but are of no theoretical interest. There are, however, a number of free parameters that do affect the action of the supervisory processes, including: generation period, controlling the number of cycles between responses; Working Memory decay, which was related to the per-item capacity of Working Memory; and parameters controlling the likelihood of updating Working Memory and of the Monitoring process firing. Such parameters and their values are given in the Supplementary Material.

In order to establish baseline performance, model parameters were hand-set such that the model fit human performance in a single empirical condition. Simulations then consisted of systematically manipulating only the generation period parameter, which was hypothesized to produce similar modulation of randomness indices to varying generation interval in human subjects. This procedure was adopted so as to avoid arbitrary model performance produced by atheoretically fitting free parameters. For qualitative examination of model behavior, fine-grained variations of the generation period parameter were used to graph how biases in random sequences varied as a function of generation rate. For quantitative analysis, six regularly-spaced levels of this parameter were assumed to correspond ordinally to the levels of generation interval in the target data (i.e., fastest to slowest) described below. This gives the six experimental conditions for each virtual subject referred to above.

#### Target data

**Figure 2:**
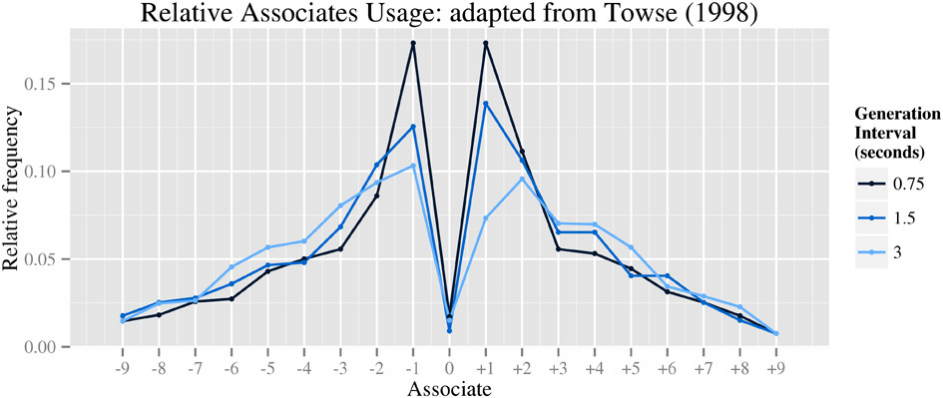
**Distribution of difference scores—the difference between consecutive responses (adapted from Towse, 1998, Figure 2)**.

The model's performance was evaluated against data from published empirical studies of RNG in human subjects. Specifically, the model aims to capture the qualitative pattern of bias modulation when generation rate is varied, which we argue reflects rate-limitations in executive processes. Unfortunately, while the behavioral effects of varying generation rate are qualitatively robust, dependent variables have not been reported consistently across published studies, making direct quantitative comparison between studies problematic. However, at least two behavioral studies report sufficient indices of randomness to infer the modulation of underlying biases. Jahanshahi et al. (2006) reported 11 varied randomness indices in a behavioral replication of previous studies (Jahanshahi et al., 1998, 2000). Additionally, Towse (1998) reports the results of statistical tests for the indices RNG, R, and A, with findings consistent with the Jahanshahi studies. The qualitative patterns of bias modulation are as follows (see Table 2). Firstly, both studies report a main effect of generation rate on measures of consecutive associates (CS1, A), with faster rates of generation significantly more biased than slower rates. Secondly, finer-grained analysis of bias in associates usage (e.g., Towse, 1998, reproduced here as Figure 2) illustrates the shape of response biases that contribute to the RNG score. Features of this data include the typical overproduction of ±1 associates at fastest rates of generation (i.e., 1.33 Hz) and their suppression at intermediate (0.66 Hz) and slower rates (0.33 Hz), to the extent that +2 associates were more frequently used than +1 associates at the slowest rate. Unfortunately, formal statistical tests of these data were not reported. Thirdly, both Towse (1998) and Jahanshahi et al. (2006) report significant effects of generation rate on overall associate bias (RNG), although in Jahanshahi et al. (2006) this was restricted to an increase at only the fastest rate of generation (2 Hz), whereas in Towse (1998) the increase appeared gradual. No further effects of generation rate are anticipated.

**Table 2:** **Mean and SD for all randomness indices, target data, and simulations**.

	**Generation rate**	**(Towse, 1998), experiment 1^a^**	**(Jahanshahi et al., 2006), experiment 1^b^**	**Simulation 1A^c^**	**Simulation 1B^d^**	**Simulation 2A^e^**	**Simulation 2B^f^**
CS1	Slow		15.4 (3.6)	11.3 (5.1)	15.0 (5.7)	13.9 (5.5)	22.6 (12.2)
	Medium		28.2 (5.1)	28.1 (8.7)	22.8 (7.4)	29.7 (12.0)	28.3 (12.4)
	Fast		48.9 (4.6)	58.5 (2.8)	56.7 (16.6)	56.6 (25.7)	57.9 (17.5)
CS2	Slow		27.5 (3.1)	29.6 (6.6)	30.3 (8.4)	32.1 (7.9)	28.2 (8.4)
	Medium		33.3 (3.5)	27.7 (5.6)	32.4 (9.0)	30.0 (16.0)	35.4 (16.3)
	Fast		30.1 (1.8)	22.6 (7.9)	24.1 (7.1)	23.9 (10.0)	25.4 (11.2)
R	Slow	1.03		1.06 (0.40)	1.20 (0.64)	2.52 (1.04)	2.48 (1.40)
	Medium	1.24		1.26 (0.72)	1.19 (0.68)	2.04 (1.07)	2.12 (1.22)
	Fast	2.10		1.11 (0.67)	0.91 (0.37)	1.45 (0.73)	1.50 (0.68)
RNG	Slow	0.255	0.332 (0.009)	0.278 (0.026)	0.260 (0.026)	0.335 (0.031)	0.325 (0.037)
	Medium	0.292	0.319 (0.011)	0.257 (0.017)	0.255 (0.027)	0.286 (0.035)	0.294 (0.037)
	Fast	0.328	0.349 (0.011)	0.279 (0.029)	0.271 (0.030)	0.279 (0.022)	0.287 (0.040)
RG	Slow		7.8 (0.1)	7.5 (0.6)	7.9 (0.5)	7.4 (0.5)	7.7 (0.8)
	Medium		8.0 (0.2)	7.4 (0.6)	7.6 (0.4)	7.3 (0.5)	7.4 (0.7)
	Fast		7.6 (0.2)	7.9 (6.7)	7.6 (0.4)	7.8 (0.4)	7.7 (0.7)

a Generation intervals: 3, 1.5, 0.75 s. Raw scores obtained from standardized scores presented in Table 1 by scaling against expected values obtained from a pseudorandom algorithm.

b Generation intervals: 3, 1.5, 0.5 s. Data extracted from Figure 1 of Jahanshahi et al. (2006), corrected for set-size to facilitate direct comparison with other data sets.

c Generation intervals (model cycles): 33, 18, 4.

d Generation intervals (model cycles): 33, 18, 4.

e Generation intervals (model cycles): 34, 23, 12.

f Generation intervals (model cycles): 34, 23, 12.

## Results

Means of each dependent measure (CS1, CS2, R, RNG, and RG), together with human data from the target studies, are shown in Table 2. Data for three representative conditions are presented, representing slow, medium, and fast rates of generation.

**Figure 3:**
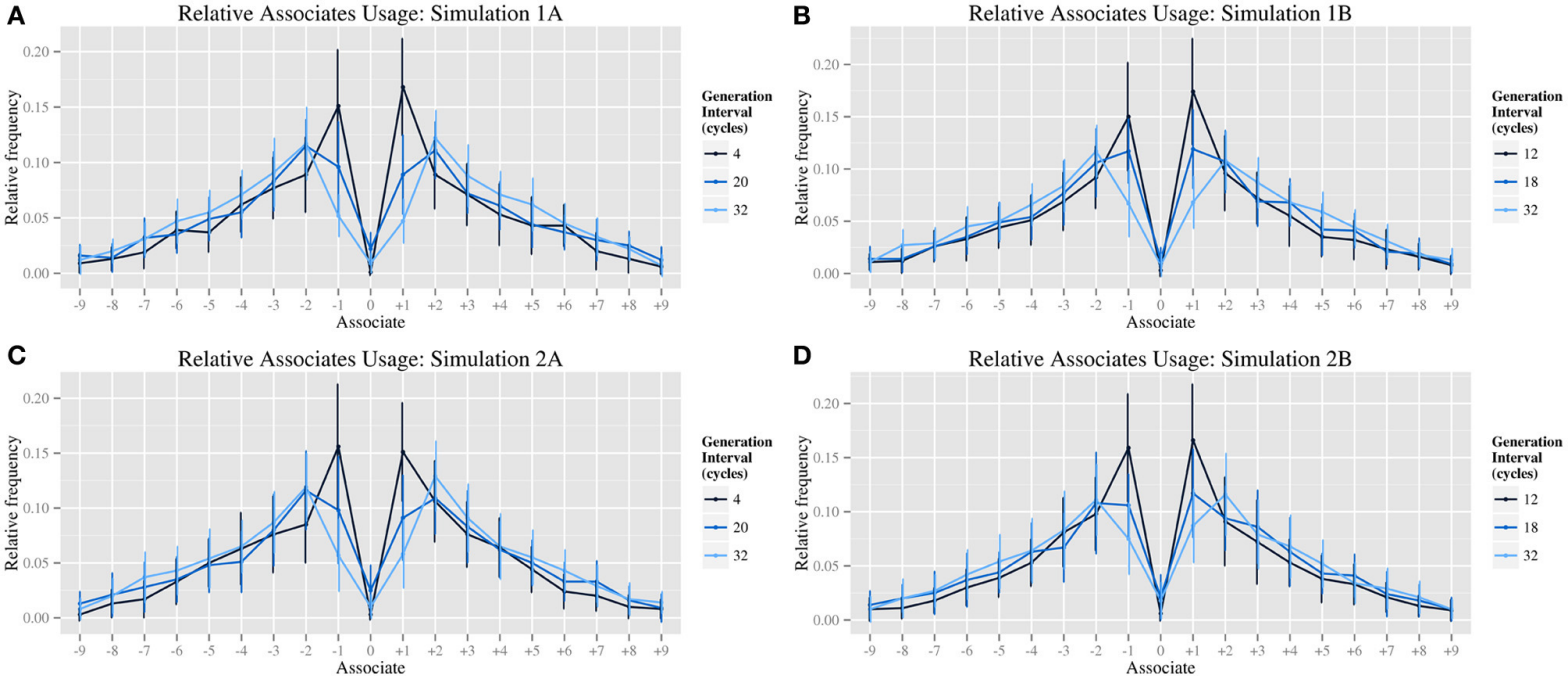
**Graphs of specific biases at varied generation rates; relative associates usage for simulations 1A (A), 1B (B), 2A (C), 2B (D)**.

The switch in bias from CS1 to CS2 at slower generates rates was assessed graphically (cf. Figure 2 for human data and Figure 3 for simulation data). All simulations reproduced the key effect, with ±1 associates being more frequent than ±2 associates at fast generation rates and *vice versa* at slower generation rates. For the remaining dependent measures, one-way independent measures ANOVAs were performed to test the effect of generation period across the six generation rates. Minor effects of skew were disregarded as the sample size was generous (*N* = 36). In cases of nonhomogeneous variances, the test reported is Welch's *F*.

Consistent with the target data, the effect of generation rate on CS1 was highly significant in all four simulations, with a very large effect size [Simulation 1A: *F*_(5, 94.47)_ = 82.43, *p* < 0.0001, η^2^ = 0.64; Simulation 1B: *F*_(5, 96.54)_ = 65.16, *p* < 0.0001, η^2^ = 0.71; Simulation 2A: *F*_(5, 95.53)_ = 74.62, *p* < 0.0001, η^2^ = 0.65; Simulation 2B: *F*_(5, 97.278)_ = 29.85, *p* < 0.0001, η^2^ = 0.54]. Also consistent with the target data, the effect of generation rate on CS2 was significant, but with a much smaller effect size reflecting a more modest increase at intermediate rates [Simulation 1A: *F*_(5, 210)_ = 5.42, *p* < 0.0005, η^2^ = 0.11; Simulation 1B: *F*_(5, 210)_ = 4.59, *p* < 0.001, η^2^ = 0.10; Simulation 2A: *F*_(5, 210)_ = 3.60, *p* < 0.005, η^2^ = 0.08; Simulation 2B: *F*_(5, 210)_ = 2.68, *p* < 0.05, η^2^ = 0.06]. Thus, the effect of generation rate on CS1 and CS2 appears robust across all four simulations, suggesting it is independent both of the specific working memory implementation (i.e., simulations 1 and 2), and to whether the distribution of schema selection probabilities is fixed (i.e., simulations 1A and 2A) or subject to individual variation across subjects (i.e., simulations 1B and 2B).

More equivocally, the main effect of generation rate on R and RNG varied between simulations. For R, the main effect of generation rate was significant but with a small effect size in Simulation 2B [*F*_(5, 210)_ = 4.60, *p* < 0.001, η^2^ = 0.08] and Simulation 2A [*F*_(5, 210)_ = 6.16, *p* < 0.0001, η^2^ = 0.13], but not in Simulation 1A [*F*_(5, 97.227)_ = 0.581, *p* = 0.71], or Simulation 1B [*F*_(5, 210)_ = 1.49, *p* = 0.20]. In both simulations in which a significant effect was shown, the value associated with ideal randomness lies within 1 SD of the mean in nearly every condition, although an overall trend is evident in simulation 2A, for less equal usage of responses at slower rates of generation. For RNG, the main effect of generation rate was significant in Simulation 1A [*F*_(5, 97.18)_ = 6.01, *p* < 0.0001, η^2^ = 0.11], Simulation 2B [*F*_(5, 210)_ = 14.06, *p* < 0.0001, η^2^ = 0.25] and Simulation 2A [*F*_(5, 97.24)_ = 31.64, *p* < 0.0001, η^2^ = 0.45], but not in simulation 1B [*F*_(5, 210)_ = 1.69, *p* = 0.14]. An overall trend toward more biased digram usage at slower rates of generation is evident in simulations 2A and 2B only. As predicted, between-subjects variability affected the RNG score, however rather than producing a uniform effect, between-subjects variability appeared to interact with generation rate, with a trend toward greater bias at slower rates of generation in B simulations but not A simulations. Thus, R and RNG both appear somewhat dependent on between-subjects variability in schema selection. For R, a small but statistically significant effect was observed in B simulations only. For RNG, significant effects with a larger effect size were observed in B simulations, and in A simulations the effect was either not significant (2A) or significant but with a smaller size of effect (1A).

Finally, the effect of generation rate on RG was significant, with large effect size in Simulation 1B [*F*_(5, 210)_ = 7.16, *p* < 0.0001, η^2^ = 0.38] and small effect size in Simulation 1A [*F*_(5, 210)_ = 2.80, *p* < 0.05, η^2^ = 0.06], Simulation 2A [*F*_(5, 210)_ = 3.56, *p* < 0.005, η^2^ = 0.08] and Simulation 2B [*F*_(5, 210)_ = 6.25, *p* < 0.0001, η^2^ = 0.13].

## Discussion

### Evaluation of model performance

The simulations reproduce the main empirical effect of varying generation rate reported by Jahanshahi and colleagues: a statistically significant modulation of CS1 score with a large effect size, reflecting increased use of a “counting” strategy at faster generation rates. The model also produces a significant effect of generation rate on CS2 in all four simulations, with much smaller effect sizes for this measure. While Jahanshahi et al. (2006) did not report such an effect, their data does show a corresponding non-significant trend. As all three studies constituting target data use a relatively small sample size (between 6 and 15 subjects), they may lack sufficient power to detect real but small effects, thus we postulate that this effect would be significant in a behavioral replication with a larger sample size. Qualitatively, the model produces a distribution of associates that matches the pattern of data in human subjects, (Figure 3) including a shift from ±1 as dominant associates at the fastest rates, to a dominance of ±2 associates at the slowest rates. This is achieved by manipulating only the equivalent model parameter, which controls the number of cycles of processing between responses. Interestingly, modulation of generation rate in simulations 1B and 2B produces a non-monotonic modulation of CS2—the apparent use of ±2 schemas is greatest at intermediate rates of generation. In the model, this occurs as a result of a sequential process. A ±1 response becomes highly active and is proposed as a putative response. This putative response is inhibited by the monitoring system before it is produced, but meanwhile activation has spread to the ±1 response's next neighbor. As spreading activation is bistable, tending to continue spreading in a single direction (i.e., counting either up or down) the next generated response is reasonably likely to be a ±2 response. The time required to complete such a sequence tends to match the period available at intermediate rates of generation, thus making ±2 associates (counting in twos) slightly more likely than other associates. This explanation has a clear analog with the explanation given by Jahanshahi et al. (2000)—given more time for each response, subjects suppress counting in ones, in favor of counting in twos. The peak in CS2 score matches a non-significant trend reported in Jahanshahi et al. (2006), and is a prediction that this will reach significance with a larger number of subjects.

Despite the model's success in simulating a distribution of associates consistent with the target data, simulations 1A and 1B fail to produce RNG scores that are in a characteristically human range. Yet, the RNG score is an overall measure of associate bias, reflecting bias away from uniform digram usage. Such a bias would result from preferred continuations (e.g., a tendency to follow “3” with “8”). It may seem, therefore, that the simulations' unrealistic RNG scores in simulations 1A and 1B are inconsistent with the good fits of the associates graphs. How can they be reconciled?

The hypothesis explored in simulations 2A and 2B is that such selection biases are indeed substantial, but vary across individuals. Consequently, idiosyncratic biases disappear from specific associates scores when averaged across participants. In the absence of variance statistics in the target data it is impossible to ascertain to what extent the qualitative shape of the associates bias is representative of an individual subject or a group average. Additionally, associates scores (e.g., +2) are themselves a composite of paired responses (e.g., 1–3, 2–4, 3–5), and variance in the relative preference of different continuations indexed by the same associate (e.g., a bias for 3–5 but against 5–7) would contribute to a higher RNG index but not a +2 associate score. Hence, while individual variability in certain biases may be either invisible in associates scores, or averaged out across participants in a group mean, bias as measured through RNG scores is neither. If this is the case, it is not appropriate to use a model with fixed associate biases to model such an index of empirical data obtained from a large sample.

Indeed, generation rate has a much larger effect on RNG in simulations 2A and 2B than in simulations 1A (where the effect was significant, but small) and 1B (where the effect was not significant). Thus, simulations 2A and 2B demonstrate that typical human values of RNG imply a greater degree of individual bias than observed in published graphs of sample means (e.g., Figure 2). While such graphical representations illustrate commonalities in biases across a population, such as the habitual use of counting, fully assessing the biases of individuals within a larger sample requires measures such as the RNG index. This finding has methodological implications for how bias is measured in empirical studies using the RNG task.

In these simulations, however, the RNG index was modulated by generation rate in an opposite direction to the human data. Thus, these simulations do not support the suggestion that in human subjects, the modulation of the RNG index is a direct effect of a variable interval in which to propose and evaluate responses for randomness, as tested in these simulations. Instead, we speculate that the modulation of the RNG index may reflect biases in Task Setting which are a result of increased cognitive load, and are thus an indirect, rather than a direct, effect of manipulating the generation rate. Incorporating a task selection bias (e.g., a bias toward more well-rehearsed schemas) mediated by cognitive load would be within the scope of the proposed architecture, but remains a hypothesis to be explored in future empirical and computational modeling research.

For R, similarly to RNG, between-subjects variability in schema selection bias (i.e., simulations 2A and 2B) interacted with generation rate to produce a slight modulation in bias. However, we recall that while Towse (1998) found no effect of generation rate on redundancy, early RNG studies report increased redundancy at faster rates, the opposite direction of effect to the simulations (e.g., Baddeley, 1966). Given that R reflects preference for specific responses, it seems likely to reflect idiosyncratic biases similar to those for digram usage. Such biases could be modeled by adding noise to individual Response Nodes' self-excitation weights, so each subject has a preference for the activation of certain response nodes (such an arrangement is suggested by Jahanshahi et al., 1998) however without more robust behavioral data to constrain modeling, we have not attempted this simulation in the present study.

### Model significance and relation to verbal models of RNG

To date, we are not aware of other mechanistic computational accounts of cognitive processes underlying RNG, hence we cannot quantitatively evaluate model performance in relation to other models. The operation of the model can be qualitatively compared to verbal models of RNG, however.

Essentially, the model operationalizes the theoretical proposal that the generation of random responses involves the suppression of the most habitual responses, counting in ones (Jahanshahi et al., 1998, 2000). At slower rates, the model tends to count in twos as a result of the proposal, and subsequent suppression, of ±1 responses before production. However, while Jahanshahi and colleagues implied that selection occurs in parallel, with a shift in CS1/2 bias at fastest rates a consequence of a breakdown of modulatory influence in the DLPFC; in the model reported here the evaluation and selection of responses takes place serially, in a temporally extended sequence. A serial mechanism has the benefit of explaining offset peaks of CS1/CS2 scores at different generation rates. Under faster rates of generation, there is no breakdown of modulatory influence—the consequence of faster rates of generation is an increasingly truncated representation of previous responses in working memory due to longer retrieval times of older (less active) items, and thus a progressive impairment in monitoring effectiveness.

The verbal process model of Baddeley et al. (1998) suggested that schema-based generation of responses is modulated by inhibition of immediate responses together with a monitoring system that detects and inhibits inappropriate overuse of schemata. This is a reasonable broad description of the model operation. In the model, the operation of the supervisory components (including the mechanisms of monitoring, inhibition of proposed responses, switching generation schema, and activating and proposing an alternative) is constrained by time available for each generation, which limits the number of possible alternatives at faster rates. While Baddeley and colleagues suggested that the schema switching process was the source of capacity limitations, in the present model the complex behavior of inhibiting an inappropriate response and proposing an alternative requires time for the interaction of a number of processes, including: checking the response against working memory; inhibiting a proposed response; activating an alternative response node; set switching; and generating a response from a novel schema. While it has been suggested that the concept of randomness is complex and varies between subjects, the model implements a rudimentary set of rules used by the monitoring system (informally, inhibiting recently generated responses, inhibiting responses generated by recently used schemas, and inhibition of counting) supporting the idea that a sophisticated conception of randomness is not necessary to produce qualitatively human-like behavior. In summary, previous verbal models of the RNG task are consistent with the model, and the favored ESPro architecture, although the latter emphasizes the interactive nature of multiple, computationally heterogeneous executive functions.

We make no strong theoretical commitment to how the concept of randomness is represented semantically. Nevertheless, the existence of such a concept is assumed by our model: one role of the monitoring system is to implement such a concept over successive digit productions. While such a concept may plausibly vary between individuals in sophistication, compared to a normative standard, the model shows that abstracting such a concept in terms of simple if/then production rules is sufficient to account for a wide range of observed human biases. One area for future research might be to evaluate various theories of randomness concepts in the model, by changing the rules implemented by the monitoring system and comparing behavioral predictions. We believe one advantage of architectural approaches in general is to facilitate such tests of theory by providing a computational framework for generating such predictions.

While the RNG task is of intrinsic interest as an index of executive function, it has been argued here and elsewhere, e.g., that the suppression of stereotyped, habitual behavior in favor of controlled, deliberately selected sequences of action is characteristic of a wide range of naturalistic human behavior as well as behavior on complex psychological tasks. From this perspective, RNG is paradigmatic of complex, self-directed and non-routine behavior. The model presented here demonstrates that such behavior can be produced by a functionally decomposed, well-specified, set of mechanistic processes without recourse to an “homunculus.”

The model thus demonstrates the viability of modeling complex “executive” tasks as the integrated operation of computationally heterogeneous processes. However, we regard the ESPro architecture outlined in this model as preliminary. The model invokes two mechanisms of convenience: setting up and realizing intentional markers (which is the proper role of further supervisory processes, hitherto explored in a somewhat separate line of research), and the role of Monitoring in suppressing inappropriate, out-of-bounds responses (e.g., responding “25” when the task is to produce random numbers ranging from “0” to “9”). Such responses are occasionally made by normal human subjects. These errors are reminiscent of certain errors of patients with prefrontal lesions on naturalistic and lab-based tasks (Shallice and Burgess, 1991). However, such errors in the RNG task have not, to our knowledge, been systematically studied, in either normal human participants or frontal lesion patients. Given the lack of quantitative data on out-of-bounds rule-breaks, this phenomenon has not been addressed here, but it may indicate a fruitful paradigm for clarifying the computational functions of the monitoring process.

While the simulations demonstrate that model behavior is largely independent of working memory implementation, both sets of simulations assume that working memory decay is a function of the number of intervening items, rather than time, yet this remains to be explained. A possible mechanism was proposed to be rehearsal as a by-product of memory retrieval by a monitoring process, however this remains to be demonstrated within the model. Indeed, a complete specification of a supervisory system architecture should clearly specify working memory rehearsal processes where they integrate with supervisory processes.

### The ESPro cognitive architecture

It was argued above that one advantage of developing a model within a cognitive architecture was that when models of a range of tasks are developed within a single architecture, constraints from the full range of tasks could help to further specify elements of the architecture, thereby effectively resulting in additional constraints on each specific model. Yet the ESPro architecture has not as yet been applied to a wide range of tasks (but see Sood and Cooper, 2013, for an implementation of the Wisconsin Card Sorting Task in essentially the same architecture). What is the evidence in support of the ESPro architecture and why did we choose to implement the RNG model within this rather than within a more widely used cognitive architecture such as ACT-R?

The primary evidence in support of ESPro is neuropsychological. The gross organization of the architecture is based on theories of cognitive structure developed on the basis of regularities in the behavior of patients with focal brain injury. In particular, patients with focal frontal lesions tend to perform well on routine tasks, where schema-based responding is adequate (Luria, 1966; Shallice, 1988). A second source of evidence for ESPro comes from cognitive psychological studies of executive function, and in particular claims of separable or fractionable executive functions such as set-shifting, response inhibition and memory monitoring/maintenance (e.g., Miyake et al., 2000). ESPro combines insights from these two areas in a way that is very different from other cognitive architectures, many of which are developed by extrapolating single domain theories to wider cognition. Thus, the Soar cognitive architecture is founded on the assumption that all cognition is a form of problem solving (Newell, 1990), while the roots of ACT-R lie in human associative memory (Anderson and Bower, 1973). While one could no doubt develop a model of RNG within, for example, Soar or ACT-R, it is unclear how the architectural assumptions of such systems would constrain or inform such a model. Doing so would essentially treat the production system base of Soar or ACT-R as a general programming language within which to implement the model. In contrast ESPro provides constraints on the RNG model from the above cited neuropsychological and cognitive psychological studies. The ESPro architecture may therefore complement such existing architectures, by providing a framework for exploring cognitive control (i.e., “executive”) functions, in terms of computationally well-specified processes with distinct, cortically localizable neural substrates.

### Conflict of interest statement

The authors declare that the research was conducted in the absence of any commercial or financial relationships that could be construed as a potential conflict of interest.

## Footnotes

^1^We take an erroneous concept of randomness to include both biases reflecting lay-beliefs about randomness (e.g., the gambler's fallacy), or due to ill-specified instructions (see Nickerson, 2002 for a review of both issues).

^2^The simulations described in this paper were written in C, using the GTK+ graphical toolkit for interface functions. All model code and simulated data are available from the authors. To compile and run the simulations GCC version 3.0 and GTK+ version 2 or similar are required. Both are freely available with any modern GNU/Linux distribution.

^3^It is plausible, however, that limitations in applying a concept of randomness to an extended sequence, in a perception task, is subject to the same capacity limitations as sequence production, and we regard this interpretation as essentially compatible with the model of production presented here.

^4^More generally, Monitoring may also make use of a forward model (not shown in Figure 1) to compare anticipated sensory (including proprioceptive and perceptual) feedback with actual sensory feedback. While this form of monitoring is important within the generalized ESPro architecture it is elided here for reasons of simplicity.

^5^The architecture as shown in Figure 1 includes three other buffers. Response Buffer and Current Set are assumed for the purposes of this implementation to be capable of holding exactly one element. It is moreover assumed that decay does not operate on these buffers. Current Set is a proxy for the schema network of Cooper and Shallice (2000) implementation of contention scheduling, with the contents of Current Set corresponding to the selected schema within that implementation. The current simulations do not make use of sensory feedback, so the properties of Sensory Stores are not relevant here.

^6^Note that while the proposed response is inhibited, it remains in the Response Buffer. Thus, it could still be generated if the generation period expires, but another response has not yet been proposed.

^7^The COGENT framework offers a range of off-the-shelf buffer implementations via parameters specifying access, capacity and decay characteristics suitable for implementing paradigmatic storage components (such as short-term limited capacity components and long-term unlimited capacity components; see Cooper, 2002).
