# Supplementary material for: An architecturally constrained model of random number generation and its application to modeling the effect of generation rate
Source: Front Psychol. 2014 Jul 1;5:670. doi: 10.3389/fpsyg.2014.00670 (PMC4076660; doi:10.3389/fpsyg.2014.00670)
Supplement: Supplementary file 1 [file DataSheet1.PDF]

Appendix: Further details of the model

I. Model Parameters

a. Monitoring

The monitoring process is assumed to operate stochastically, with the probability of operating on any cycle given by an efficiency parameter. This was set to 0.09 for all simulations.

b. Task Setting

Task setting detects whether behaviour is appropriately governed by a schema simply by regularly checking that a response is being proposed in the response buffer. If, on checking, no response is present, the current schema is cleared, triggering a process of schema selection. Checking occurred every 6 cycles for all simulations.

c. Response Nodes

Spreading activation between response nodes was implemented as an interactive activation network. Each node's input was connected to the output of all other nodes with distinct weight values for the next node (+1), the previous node (-1), and all other nodes (cf. Table 3). While the parameter values were hand-set, the overall operation of response nodes was constrained by two requirements (derived from Jahanshahi et al., 2000). Firstly, a fully active response node should inhibit all others such that only a single node may be fully active at a time (i.e., winner-takes-all). Secondly, without outside modulation, response nodes should produce counting forwards or backwards. The present implementation produces a bistable pattern where sequential response node activation occurs either forwards or backwards depending on starting activations, although with a slight bias toward counting forwards.

Table 1: Response node connection weights

**Connection**	**Weight**
Previous response node	-0.0239
Self (recurrent)	0.5000
Node 9 to 0	-0.0247
Node 0 to 9	-0.0252
Next response node	-0.0241
All other nodes (inhibitory)	-0.0330

On each cycle, the new activation of each node was calculated according to the sigmoid activation functions given in Appendix A of Cooper and Shallice (2000), from the total input excitation, using a persistence parameter which was set to 0.93 for all simulations. Response node initial activation was given by a uniform random distribution between 0.0 and 0.3. Response nodes were considered active on passing an activation threshold of 0.80. Response nodes were updated iteratively 14 times on every model cycle.

Response node activity was modulated by other model processes by way of EXCITE and INHIBIT messages. A message is translated into activation using the following scaling parameters:

Input to response nodes from EXCITE message: 0.18

Input to response node from INHIBIT message: -8.00

Note that EXCITE messages persist until they are cleared by another process, whereas INHIBIT messages only persist for a single cycle, hence a much stronger scaling value is used for INHIBIT messages.

II. Working memory activation equations

a. Simulations 1A and 2A

The activation of new items added to the buffer, A_i, is calculated as the sum of half of the activation of all other items in the buffer:

(1) $$A_i = \sum_j \frac{A_j}{2} + \left(1 - \sum_j A_j\right)$$

The activation of all other items in the buffer is then updated from A_{old} to A_{new}:

(2) $$A_{new} = \frac{A_{old}}{2}$$

Thus, new items effectively 'steal' half the activation of other items in the buffer, in addition to a 'top up' such that the total activation in the buffer is equal to 1. The activation of an item in working memory therefore tends to be approximately equal to the inverse of its position in the buffer, where the most recent item is in position 1.

Items decay (i.e., are completely removed from the buffer) when activation falls below a threshold, τ, given by equation 0:

(3) $$\tau = \frac{1}{1+d} + r$$

Where d is a working memory decay parameter, and r is a a noise value, given by a gaussian distribution centred on 0 with standard deviation $(1+d)^{-1}$. In simulations 1A and 2A, d was fixed to 40.

b. Simulations 1B and 2B

Under implementation 1B and 2B, items in WM are regarded as activated portions of long-term or semantic memory. This buffer contains a representation of all items in the response set (reflecting a semantic representation of each response), each of which has an associated activation value.

The activation A_i of items in WM is given by the base-level learning equation in ACT-R, where t_k is the time since the kth activation of an item, and d is a free decay parameter of the model (Anderson, 2007, p. 110):

(4) $$A_i = \ln\left(\sum_{k=1}^{n} t_k^{-d}\right)$$

As equation (4) becomes computationally intensive as the number of activations of an item (k) becomes large, a computationally efficient approximation to this equation was used, with k = 1, as suggested by Petrov (2006):

(5) $$A_i \approx \ln\left[\sum_{i=1}^{k} t_i^{-d} + \frac{(n-k)\left(t_n^{1-d} - t_k^{1-d}\right)}{(1-d)(t_n - t_k)}\right]$$

The decay parameter, *d*, was set to the default in ACT-R of 0.5.

The probability of successfully retrieving an item from WM, *p*, is a given by the ACT-R retrieval probability equation, (6), where A_i is the activation of an item, τ is a threshold parameter, and *s* is a noise parameter (Anderson, 2007, p.110). For simulations 1B and 2B, τ was fixed at 0.0 and s was fixed at 1.0.

(6) $$p = \left(1 + e^{\frac{-(A_i - \tau)}{s}}\right)^{-1}$$

The model diverged from a standard ACT-R implementation of declarative memory. When calculating the retrieval probability equation and the retrieval latency equation, the activation of the item retrieved, A_i was assumed to be the difference between an item's absolute activation and the mean activation of *n* items in the buffer.

(7) $$A = A_i - \frac{1}{n}\sum_{j=1}^{n} A_j$$

III. References

Anderson, J. R. (2007). *How Can the Human Mind Occur in the Physical Universe?* New York, NY: Oxford University Press. doi: 10.1093/acprof:oso/9780195324259.001.0001

Cooper, R., and Shallice, T. (2000). Contention scheduling and the control of routine activities. *Cogn. Neuropsychol.* 17, 297–338. doi: 10.1080/026432900380427

Jahanshahi, M., Dirnberger, G., Fuller, R., and Frith, C. D. (2000). The role of the dorsolateral prefrontal cortex in random number generation: a study with positron emission tomography. *Neuroimage* 12, 713–725. doi: 10.1006/nimg.2000.0647

Petrov, A. (2006). "*Computationally efficient approximation of the base-level learning equation in ACT-R,"* in *Proceedings of the Seventh International Conference on Cognitive Modeling*, eds D. Fum, F. Del Missier and A. Stocco (Trieste, Italy: Edizioni Goliardiche).
